# Post-acute withdrawal syndrome (PAWS) after stopping antidepressants: a systematic review with meta-narrative synthesis

**DOI:** 10.1017/S204579602500023X

**Published:** 2025-05-13

**Authors:** Andri Rennwald, Michael P. Hengartner

**Affiliations:** 1 School of Applied Psychology, Zurich University of Applied Sciences, Zurich, Switzerland; 2 Department of Applied Psychology, Kalaidos University of Applied Scieces, Zurich, Switzerland

**Keywords:** antidepressant, discontinuation, harm, persistent, post-acute, protracted, safety, withdrawal

## Abstract

**Aims:**

The literature on persistent antidepressant withdrawal symptoms is sparse. This systematic review is the first to examine the prevalence, duration, severity, risk/protective factors and treatment strategies for post-acute withdrawal syndrome (PAWS) following the discontinuation of antidepressant medications.

**Methods:**

We searched PubMed, Web of Science and PsycInfo, focusing on newer-generation antidepressants. The electronic database search was complemented with handsearching reference lists of pivotal studies. We included original studies in adults reporting on PAWS and providing data about epidemiology and clinical management of withdrawal symptoms persisting for at least 6 weeks.

**Results:**

The literature search yielded 1286 results, with 26 records assessed for eligibility, and seven studies fulfilled our selection criteria. Prevalence data were sparse, with one small cohort study reporting a 15% prevalence rate for PAWS in patients with panic disorder and agoraphobia. The duration of PAWS varied considerably across studies, ranging from 1.5 to 166 months. Long-term paroxetine use emerged as a potential risk factor for the development of PAWS. There was no reliable evidence to support the effectiveness of various treatment strategies, including the reinstatement of antidepressant medication, the use of benzodiazepines and the provision of cognitive-behavioral therapy.

**Conclusions:**

The current evidence on PAWS is sparse and predominantly of low certainty. The presence of withdrawal symptoms, lasting several months and possibly even years in some patients, underscores the need for further research with rigorous methodology. Large prospective cohort studies are needed to assess the epidemiology of PAWS, while randomized controlled trials are quired to test the efficacy of clinical interventions to treat PAWS.

**PROSPERO registration:**

CRD42023461793

## Introduction

Growing long-term use, often over several years, is a significant reason for the world-wide increase in the consumption of antidepressant medications over the last 2–3 decades (Aarts *et al.*, 2014; Amrein *et al.*, 2023; Mars *et al.*, 2017). The increasing prevalence of long-term antidepressant use amplifies the importance of understanding the risks associated with long-term use. One recently identified main concern is the potential for acute and persistent post-acute (protracted) withdrawal symptoms when the medication is discontinued (Boland *et al.*, 2024; Chouinard and Chouinard, 2015; Fava and Cosci, 2019; Hengartner *et al.*, 2019; Lerner and Klein, 2019; Massabki and Abi-Jaoude, 2021). The prolonged use of antidepressant medications, similar to other psychotropic substances, can lead to sustained neurophysiological adaptations (Ashton, 1991; Cosci and Chouinard, 2020; Fava and Cosci, 2019; Horowitz *et al.*, 2023; Lerner and Klein, 2019). In basic pharmacology and addiction medicine, these neurophysiological adaptations are typically defined as physical dependence (no to be confused with addiction) and a withdrawal syndrome upon dose reduction or treatment discontinuation is its sole clinical manifestation (Horowitz *et al.*, 2023; Horowitz and Taylor, 2023; Lerner and Klein, 2019).

Withdrawal symptoms can be classified into sensory symptoms, disequilibrium, general somatic symptoms, sleep disturbances, gastrointestinal symptoms and affective symptoms (Haddad and Anderson, 2007). The most common symptoms include dizziness, vertigo, tremor, nausea, insomnia, fatigue, mood dysregulation, anxiety, panic, irritability and agitation (Fava *et al.*, 2015; Haddad and Anderson, 2007; Jha *et al.*, 2018). Serious symptoms may also include suicidal ideation and behaviour (Bloch *et al.*, 1995; Kostic *et al.*, 2024; Tint *et al.*, 2008). The literature further distinguishes between two broad categories of withdrawal syndromes: acute withdrawal syndromes, including rebound disorders, and persistent post-acute withdrawal syndromes (PAWS), including persistent post-withdrawal disorders (Chouinard and Chouinard, 2015; Cosci and Chouinard, 2020; Lerner and Klein, 2019). The acute withdrawal syndrome presents with qualitatively new symptoms unrelated to the original condition, emerging rapidly after discontinuation or dose reduction and lasting between 1 and 6 weeks. A specific subgroup of the acute withdrawal syndrome is the rebound disorder, where previously known symptoms reoccur with greater intensity (Cosci and Chouinard, 2020; Fava and Cosci, 2019). PAWS includes new symptoms as well as original symptoms but with increased intensity, appearing days or weeks, sometimes even months, after treatment discontinuation and lasting from over a month to years (Chouinard and Chouinard, 2015; Hengartner *et al.*, 2020; Lerner and Klein, 2019). Phenomenologically, the syndrome often resembles relapses of the original condition or new emergent mental disorders, as outlined by the concept of persistent post-withdrawal disorders (Cosci and Chouinard, 2020; Cosci *et al.*, 2024). To increase the inclusiveness and exhaustiveness of this review, and in accordance with previous work (e.g. Hengartner *et al.*, 2020; Lerner and Klein, 2019), we conceive of persistent post-withdrawal disorders as a manifestation of PAWS.

Acute withdrawal syndromes have been reported over decades in various case reports (e.g. Bloch *et al.*, 1995; Kostic *et al.*, 2024; Kramer *et al.*, 1961) and were later confirmed in placebo-controlled treatment-interruption trials (e.g. Michelson *et al.*, 2000; Rosenbaum *et al.*, 1998), and systematic reviews (e.g. Davies and Read, 2019; Fava *et al.*, 2015, 2018). However, there is a notable lack of research on PAWS, and to the best of our knowledge, the sparse evidence base on persistent withdrawal reactions is mostly comprised of case reports and opinion papers. The aim of this study was thus, for the first time, to systematically search the scientific literature for original studies informing about the epidemiology and clinical management of PAWS.

## Methods

The protocol for this systematic review was pre-registered on PROSPERO (CRD42023461793) and the work was conducted according to PRISMA (Preferred Reporting Items for Systematic reviews and Meta-Analyses) guidelines (Moher *et al.*, 2009). Because formal meta-analysis was not possible (see below), we also adopted RAMSES (Realist And Meta-narrative Evidence Syntheses: Evolving Standards) recommendations (Wong *et al.*, 2013).

### Data sources

Titles, abstracts and topics were searched using the following search terms in the electronic research literature databases Pubmed, Web of Science and PsycInfo from inception of each database to January 2024: ([Antidepressants OR selective serotonin reuptake inhibitor OR SSRI OR SNRI OR citalopram OR escitalopram OR fluoxetine OR fluvoxamine OR paroxetine OR sertraline OR desvenlafaxine OR duloxetine OR levomilnacipran OR milnacipran OR venlafaxine OR vilazodone OR vortioxetine OR nefazodone OR trazodone OR reboxetine OR bupropion OR mianserin OR mirtazapine OR agomelatine] AND [Post-acute OR prolonged OR protracted OR persistent] AND [withdrawal OR discontinuation OR postwithdrawal OR post-withdrawal OR post-discontinuation] AND [symptoms OR syndrome OR disorder OR reaction]). Limits were set to adults (not older than 75 years) and humans. In addition, the reference lists and citations of initially identified articles, including relevant systematic reviews and pivotal studies (e.g., Fava *et al.*, 2015, 2018), were manually searched to identify further original studies not captured by the electronic literature search. Likewise, clinical trials were additionally searched for manually in the trial registry ClinicalTrials.gov.

### Study selection

The search was independently conducted by two investigators (A.R. and M.P.H.), with any disagreements resolved through consensus among these reviewers. Articles were included if they reported at least one case of PAWS or data on persistent or protracted antidepressant withdrawal symptoms after the discontinuation of selective serotonin reuptake inhibitors (SSRI), serotonin-noradrenaline reuptake inhibitors (SNRI) or any other newer-generation monoaminergic antidepressant such as, among others, mirtazapine, vortioxetine, reboxetine or bupropion, but excluding esketamine due to its primary action on the glutamate system. We included only original studies of following designs: case reports, observational studies (case–control and cohort studies), experimental studies, consumer surveys and analyses of online self-reports in peer-support groups that included original data informing about PAWS after discontinuation of newer-generation antidepressants.

### Data extraction

Data were independently extracted by both reviewers. Extracted data included the following study characteristics: publication date, location, study design, sample size, antidepressant class or drug, assessment of PAWS, age (mean) and sex (% men and women). For the synthesis of information about PAWS, we grouped the data into the following categories: prevalence of PAWS, duration of PAWS, severity of PAWS, risk or protective factors of PAWS and treatment strategies to mitigate PAWS. Risk of bias was rated according to the Risk of Bias in studies estimating the Prevalence of Mental Health disorders (RoB-PrevMH) by Tonia *et al.* (2023).

### Data synthesis

In consideration of the few studies, sparse data, diverse patient populations and study design disparities, a formal meta-analysis as proposed in the study protocol was not possible. We therefore conducted a meta-narrative synthesis as outlined by Wong *et al.* (2013).

## Results

**Figure 1.**
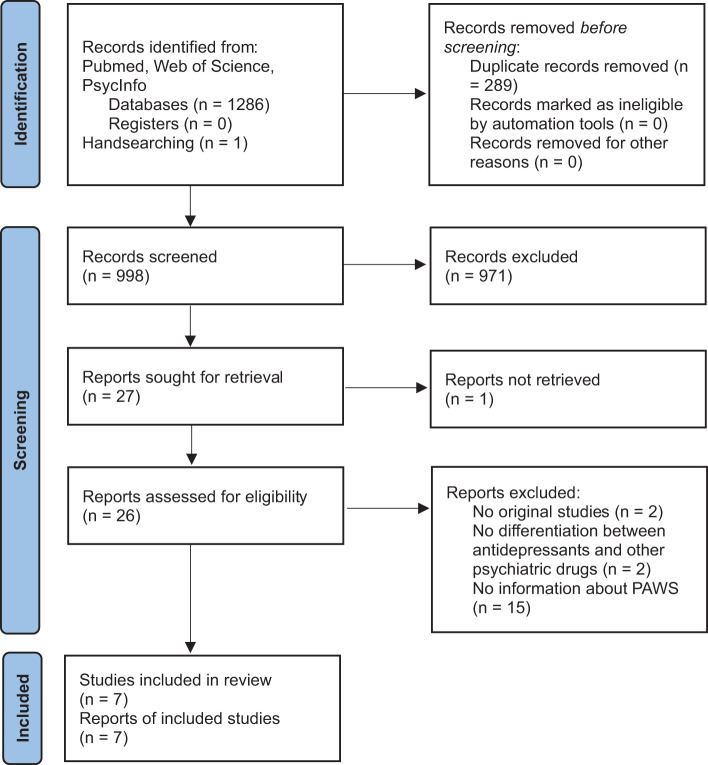
PRISMA study selection flow chart.

The initial database search generated 1286 results and handsearching of reference lists additionally captured one case report that met our selection criteria. After the removal of duplicates, 998 potentially relevant reports were left. These 998 reports were screened for abstract and title, which resulted in 27 relevant reports. Of these, one was not retrieved because it was available in Dutch only, thus 26 reports were assessed in detail. Among these, two were no original studies, two made no differentiation between antidepressants and other psychiatric drugs, and 15 did not report data informing about PAWS. We did not detect relevant articles by searching ClinicalTrials.gov. Thus, seven reports that provided pertinent data on the epidemiology and clinical management of PAWS were included in the meta-narrative synthesis (see Fig. 1).

The designs of the included studies varied greatly. There were three analyses of online self-reports in peer-support groups on the internet, one RCT, one case-series, one case report, and one prospective cohort study. Risk of bias was rated high in at least two of three domains covered in most studies. Detailed study characteristics are reported in Table 1.

**Table 1.** Description of included studies

Authors and publication date	Location	Study design	Sample size (n)	Antidepressant class or drug	Assessment of PAWS	Age (mean years)	Sex (% men, women)	Risk of bias		
								Representativeness of the sampling frame	Representativeness of the responders	Measurement of the condition
Belaise *et al.*, 2012	International	Online self-reports	12	SSRI	Spontaneous patient self-report	n.a.	n.a.	High	High	High
Belaise *et al.*, 2014	Italy	Case series	3	Paroxetine	DESS (Discontinuation-Emergent Signs and Symptoms)	40	66% male 33% female	High	High	Low
Bhanji *et al.*, 2006	Canada	Case report	1	Paroxetine	Clinical evaluation	73	Female	High	High	Low
Fava *et al.*, 2007	Italy	Prospective Cohort study	20	SSRI	DESS (Discontinuation-Emergent Signs and Symptoms)	37.7	30% male 70% female	High	Low	Low
Hengartner *et al.*, 2020	International	Online self-reports	69	SSRI SNRI	Spontaneous patient self-report	n.a.	n.a.	High	High	High
Lewis *et al.*, 2021	United Kingdom	RCT	478	Citalopram Fluoxetine Sertraline Mirtazapine	DESS (Discontinuation-Emergent Signs and Symptoms)	54	27% male 73% female	Low	High	Low
Stockmann *et al.*, 2018	International	Online self-reports	174	SSRI SNRI	Spontaneous patient self-report	n.a.	n.a.	High	High	High

### Prevalence of PAWS

Only one study provided data about the prevalence of PAWS. In this small prospective cohort study (Fava *et al.*, 2007), it was found that 3 out of 20 participants (15%), all of whom had been prescribed the antidepressant paroxetine long-term, developed PAWS after slow drug tapering. Tapering was conducted with reductions of 50 mg every other week for fluvoxamine and sertraline, and 10 mg every other week for paroxetine, fluoxetine and citalopram, followed by 10 mg every other day in the final phase before cessation. The study sample of *n* = 20 was drawn from a single outpatient clinic in Italy and the participants were all diagnosed with a panic disorder and agoraphobia, which further restricts the representativeness of this prevalence estimate.

### Onset and duration of PAWS

Several studies examined the duration of withdrawal symptoms and estimates varied greatly, ranging from about 1.5 months to many years. One analysis of online self-reports (Belaise *et al.*, 2012) reported a mean duration of 2.5 years, with a range of 0.1 to 6 years. In the other two analyses of online self-reports, duration of PAWS ranged from 5 to 166 months, with a mean of 37 and a median of 26 months according to one study (Hengartner *et al.*, 2020), and with an average duration of withdrawal symptoms of 90.5 weeks for SSRI and 50.8 weeks for SNRI in the other study (Stockmann *et al.*, 2018). A case series of three patients who discontinued paroxetine (Belaise *et al.*, 2014) reported that remission of PAWS was achieved between 3 and 5 months after the patients began treatment with cognitive-behavioural therapy (CBT). Withdrawal symptoms typically emerged within a few days of dose reduction or cessation, but delayed onset also occurred, sometimes weeks or even months after treatment discontinuation (Stockmann *et al.*, 2018). In one case report, persistent withdrawal symptoms emerged 4 weeks after abrupt discontinuation of long-term paroxetine treatment (Bhanji *et al.*, 2006). However, these estimates of symptom onset and duration are unreliable and unrepresentative, because they were mostly based on retrospective assessments in selective samples of patients presumably experiencing (or self-identifying with) more severe withdrawal symptoms. One small prospective cohort study (Fava *et al.*, 2007) reported on three patients with PAWS, in whom symptoms lasted for at least 3 months before psychopharmacological interventions led to symptom relief in two of them. In the third patient, all treatment approaches failed, and PAWS persisted beyond the 12 months of study follow-up. Finally, in a large and pragmatic real-world RCT (Lewis *et al.*, 2021), patients discontinuing antidepressants, relative to patients maintained on medication, reported statistically significantly more withdrawal symptoms after 12 weeks (3.1 vs. 1.3 symptoms), 26 weeks (1.9 vs. 1.4 symptoms) and 39 weeks (1.7 vs. 0.8 symptoms). These data provide the best evidence for the existence of PAWS, indicating that antidepressant withdrawal symptoms may persist up to at least 39 weeks in some patients.

### Severity of PAWS

Belaise *et al.* (2012) reported that patients self-identifying with antidepressant withdrawal syndrome exhibited predominantly very severe symptoms that significantly impaired their quality of life. The prospective cohort study by Fava *et al.* (2007) described a severe pattern of alternating symptoms, including worsened mood, fatigue, emotional instability, sleep disturbances, irritability, and hyperactivity in all three patients with PAWS. The syndromes met the DSM-IV criteria for cyclothymic disorder except for the required duration criterion.

### Risk and protective factors of PAWS

Two of the included studies indicate that in particular long-term paroxetine use may carry an increased risk of PAWS (Belaise *et al.*, 2012; Fava *et al.*, 2007), but most studies were uninformative on this subject. One analysis of online self-reports (Stockmann *et al.*, 2018) further showed that the duration of tapering was positively correlated with the duration of withdrawal symptoms, suggesting that patients experiencing more persistent withdrawal symptoms try to taper more slowly. In contrast, the length of prior treatment was not correlated with the duration of withdrawal symptoms, and neither length of taper nor duration of previous treatment correlated with time to onset of withdrawal symptoms (Stockmann *et al.*, 2018).

### Treatment strategies to mitigate PAWS

Four studies mentioned the reinstatement of the initial antidepressant medication or switching to a low-dose benzodiazepine as potential interventions. Belaise *et al.* (2012) noted that PAWS may last several months when the antidepressant drug is not restarted. Fava *et al.* (2007) described three patients in whom low-dose clonazepam and/or reinstitution of the original antidepressant medication helped to ameliorate the withdrawal symptoms in two of three patients. Hengartner *et al.* (2020) reported that reinstatement of the antidepressant medication resolved self-reported PAWS in 9 of 19 persons (47%) who attempted this approach. Treatment with other drugs, including benzodiazepines, pregabalin or propranolol, was attempted by 33 persons, but only 6 persons (18%) reported at least some benefit. In one case report of PAWS after abrupt paroxetine discontinuation, benzodiazepine treatment did not ameliorate the withdrawal symptoms, but reinstatement of paroxetine did (Bhanji *et al.*, 2006). One case series of three selected patients who discontinued paroxetine (Belaise *et al.*, 2014) proposed the use of CBT as a beneficial approach in managing PAWS by helping patients to cope with the withdrawal symptoms and distressing experiences. In this study, one patient achieved remission after 6 months of CBT, another experienced symptom resolution after 5 months of CBT with additional 1.5 mg/day of clonazepam, and the third had their symptoms resolved within three months of CBT (Belaise *et al.*, 2014). However, efficacy of these treatment approaches remains uncertain, since no intervention was tested in a clinical trial, and the observational studies had no control group.

## Discussion

The aim of this systematic review was to gather all relevant epidemiological and clinical information about PAWS, in particular, prevalence and duration of the syndrome, its severity, risk and protective factors, as well as potential treatment or management strategies to mitigate symptom severity and duration. Importantly, our comprehensive search detected merely seven studies that reported pertinent data and most of them provided only low-quality evidence. Three studies (Belaise *et al.*, 2012; Hengartner *et al.*, 2020; Stockmann *et al.*, 2018) were based on self-reported information posted online in peer-support groups on the Internet, such as SurvivingAntidepressants.org, by people self-identifying with an antidepressant withdrawal syndrome. Another study (Belaise *et al.*, 2014) was a case series of three patients referred to a CBT service for the treatment of PAWS after discontinuation of paroxetine and one was a case report of one patient (Bhanji *et al.*, 2006). These studies thus cannot provide reliable and representative data about the epidemiology and clinical management of PAWS. One study was a prospective cohort study (Fava *et al.*, 2007), but it included only 20 patients with panic disorder and agoraphobia treated in a single outpatient clinic in Italy. According to this study, three of 20 patients (15%) developed PAWS, and all of them had been on long-term paroxetine medication. However, given to the small sample size and the specific original treatment indication (i.e. panic disorder with agoraphobia), the estimated prevalence rate cannot be considered representative and generalizable to the broader population of antidepressant users discontinuing their medication.

Finally, one study was a large real-world RCT (Lewis *et al.*, 2021) with 478 primary care patients recruited from 150 general practices in the UK. Although the data presented did not allow the calculation of prevalence rates (requests for additional data were declined by the authors), it provides the most compelling evidence for the existence of PAWS, that is, post-acute antidepressant withdrawal symptoms persisting over several months in at least some patients. In this study, 39 weeks after patients had started tapering citalopram, fluoxetine, sertraline or mirtazapine, the number of recorded withdrawal symptoms was still significantly increased compared to patients maintained on their antidepressant medication. Of note, in the design of this RCT, the popular antidepressant drugs paroxetine and venlafaxine were deliberately excluded, because, as written by the authors, both are known to cause marked withdrawal symptoms when treatment is discontinued (Lewis *et al.*, 2021; p. 1258).

In sum, most available studies on PAWS rely on self-reported information from online support groups, such as the data examined by Belaise *et al.* (2012), Stockmann *et al.* (2018), and Hengartner *et al.* (2020). While these studies offer initial insights, they are limited by selection bias and lack of clinical validation of the original treatment indication, the drug regimen, the tapering schedule and both the timing and quality of withdrawal symptoms. The same applies to another line of research based on surveys among members of these Internet groups, including a recent study by Moncrieff et al. (2024), which came out after completion of our literature search. It confirms that people seeking help on the Internet for self-identified antidepressant withdrawal syndromes, report very high rates of persistent withdrawal symptoms. According to this particular study (Moncrieff et al., 2024), altogether 50% of help-seeking people reported withdrawal symptoms lasting more than a year, 32% more than 2 years, and 11% more than 5 years. Moreover, 61% of people reported being severely affected by withdrawal. However, due to its methodology, it cannot inform about the prevalence, severity, and course of PAWS in the broader population of antidepressant users discontinuing their medication.

Thus far, we can extrapolate from our review that antidepressant withdrawal symptoms may last for many months and even years in some patients, as previously noted in the literature (Davies and Read, 2019; Fava *et al.*, 2015; Hengartner *et al.*, 2019). The scientific evidence for these more persistent post-acute withdrawal syndromes led for instance NICE to update their antidepressant treatment guideline (Iacobucci, 2019), and the Royal College of Psychiatrists issued a position statement on discontinuing antidepressants (Royal College of Psychiatrists, 2019). Nevertheless, compared to the steadily increasing number of editorials, commentaries and narrative reviews mentioning PAWS (e.g. Chouinard and Chouinard, 2015; Davies *et al.*, 2019; Fava and Belaise, 2018; Fava and Cosci, 2019; Lerner and Klein, 2019), the lack of high-quality original studies providing reliable evidence on the epidemiology and clinical management of PAWS is striking. Large prospective cohort studies are required to accurately estimate the prevalence and severity of PAWS in the population of antidepressant users who discontinue drug treatment, and to gain insights into the timing and course of more persistent withdrawal syndromes and which clinical factors are differentially associated with them (e.g. length of treatment, dosage, tapering schedule). In addition, rigorous long-term RCTs are required to test the efficacy of treatment and management strategies. These studies would inform clinicians about effective interventions to mitigate the severity and duration of this impairing syndrome, as to date not a single clinical intervention has been formally evaluated. This accords with calls made in a recent study, according to which these clinical topics are the most important to key stakeholders such as antidepressant users and healthcare professionals (Boland *et al.*, 2024).

Unfortunately, it appears that in both clinical research and practice, protracted and persistent withdrawal symptoms are frequently misdiagnosed as a relapse of the primary mental health condition (typically depression) or new emergent mental disorders (Cosci *et al.*, 2024; Guy *et al.*, 2020; Hengartner and Plöderl, 2021), the latter being specifically embedded in the concept of persistent post-withdrawal disorders (Chouinard and Chouinard, 2015; Cosci and Chouinard, 2020; Cosci *et al.*, 2024). These issues highlight the need for more awareness and better recognition of the various manifestations of PAWS to ensure adequate patient care (Cosci *et al.*, 2024; Framer, 2021; Hengartner *et al.*, 2020; Horowitz and Davies, 2025; Read *et al.*, 2023). Finally, the etiopathology of PAWS is largely unknown, but since reinstatement of the drug quite often does not resolve the syndrome, it has been proposed that it could be caused by neural damage akin to tardive dyskinesia, irreversible pharmacodynamic processes and endocrine disruption (Fava and Cosci, 2019; Framer, 2021; Horowitz *et al.*, 2023). A promising model to guide research in this area is the oppositional model of tolerance by Giovanni Fava, which denotes PAWS as a consequence of the brain’s neurophysiological adaptation to prolonged antidepressant use, resulting in processes that oppose the acute treatment effects when users discontinue their medication (Fava, 2003, 2020; Fava and Offidani, 2011). Biological research into the etiopathological mechanisms of PAWS could thus also inform about potential prevention and treatment strategies.

## Conclusion

This systematic review with meta-narrative synthesis highlights the lack of high-quality research on PAWS, that is, persistent post-acute (protracted) withdrawal symptoms following the discontinuation of antidepressant medications. The current evidence on PAWS remains very limited and unreliable, as it is predominantly derived from a few case reports and analyses of self-reports posted on the Internet by help-seeking people self-identifying with severe antidepressant withdrawal syndromes. Based on the scientific literature, specifically emphasizing the findings of a small but rigorous cohort study (Fava *et al.*, 2007) and the results of a large RCT (Lewis *et al.*, 2021), we can merely conclude that persistent antidepressant withdrawal symptoms do certainly exist. However, based on these data we cannot estimate accurately how prevalent, persistent and impairing PAWS is. We also do not know which antidepressant users are at increased risk of PAWS, and how patients with PAWS are treated effectively. Our review’s main finding thus reflects the poor recognition and insufficient consideration of PAWS in both clinical research and practice (Cosci *et al.*, 2024; Fava and Belaise, 2018; Horowitz and Davies, 2025; Read *et al.*, 2023). To advance our knowledge of the epidemiology and clinical management of PAWS, we call for further exploration and validation through rigorous cohort studies and RCTs.

## Data Availability

All the data supporting the findings of this study can be found in this article and the reviewed reports.
